# Vine Tendril‐Inspired Cold‐Programmable Shape‐Memory Polymer Composites Using Sugar Beet Pulp

**DOI:** 10.1002/smll.74830

**Published:** 2026-07-29

**Authors:** Wei Huang, Vikramjeet Singh, Yu Wang, Junhao Chen, Mingjing Zhang, Gary J. Lye, Helen C. Hailes, Luna Jiang, Eral Bele, Manish K. Tiwari

**Affiliations:** ^1^ Nanoengineered Systems Laboratory UCL Mechanical Engineering University College London London UK; ^2^ UCL Hawkes Institute University College London London UK; ^3^ Jiangsu Key Laboratory of Advanced Food Manufacturing Equipment and Technology School of Mechanical Engineering Jiangnan University Wuxi Jiangsu China; ^4^ Manufacturing Futures Laboratory University College London London UK; ^5^ Department of Biochemical Engineering University College London London UK; ^6^ Department of Chemistry University College London London UK; ^7^ Institute of Orthopaedic and Musculoskeletal Science Royal National Orthopaedic Hospital University College London London UK; ^8^ Advanced Characterisation Lab For Materials and Manufacturing UCL Mechanical Engineering University College London London UK

**Keywords:** bio‐derived materials, bio‐inspired strategies, cold programming, shape‐memory polymers, sugar beet pulp

## Abstract

Stimuli‐responsive morphing in natural organisms enables dynamic adaptation to environmental challenges. Inspired by this, shape‐memory polymers (SMPs) can undergo controlled shape transformations under external stimuli. However, conventional SMPs, typically constructed by uniform material networks, require heat‐assisted programming to secure temporary shapes, which restricts their adaptability. By contrast, cold programming, deforming materials into a temporary shape without thermal activation, offers greater versatility but demands stringent material design. In this work, inspired by vine tendrils whose specialized cells enable asymmetric deformation (manifested macroscopically as coiling) under force, and motivated by the sustainability imperative of bio‐derived materials in reducing dependence on fossil‐based resources, we developed cold‐programmable shape‐memory polymer composites (SMPCs) incorporating bio‐based sugar beet pulp (SBP). Through a magnetically assisted technique, heterogeneous domains were constructed within the SMPCs: a formulated SMP‐rich region serving as the shape‐stabilizing phase, and an SBP‐enriched domain with a rough morphology enhancing mechanical resilience. After being stretched, the SMPCs underwent asymmetric recovery, enabling controlled actuations. This work highlights the potential of cold‐programmable, deployable structures to advance smart material functionality while providing a sustainable pathway via bio‐based components for emerging aerospace and soft robotics applications.

## Graphical abstract

Vine tendrils inspire a bio‐derived, cold‐programmable shape‐memory polymer composite (SMPC). Magnetically guided sugar beet pulp creates mesoscale heterogeneity within a glassy matrix, producing built‐in mechanical asymmetry for rapid, solvent‐free programming by simple stretching. The sustainable SMPC delivers reversible actuation and deployable, power‐independent structures, opening new possibilities for smart robotics and aerospace protection.

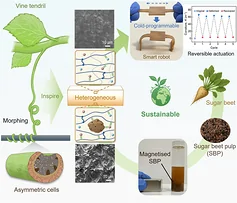

## 1 Introduction

Stimuli‐responsive shape‐morphing behaviors are commonly observed in natural organisms, including plants [1], invertebrates [2], and microorganisms [3], allowing them to adapt dynamically to environmental fluctuations and thrive in challenging conditions. This remarkable adaptability has inspired the development of artificial materials that mimic these biological behaviors, leading to advanced shape‐morphing capabilities [4, 5, 6, 7]. Among these materials, shape‐memory polymers (SMPs) are particularly notable for their unique properties, including ease of customization, a broad stiffness range, and rapid responsiveness to external excitation [8], making them highly promising for applications in biomedical devices [9, 10, 11], soft robotics [12, 13], and aerospace [14, 15].

Typically, SMPs can be programmed into a temporary shape that can effectively return to their original shape when exposed to heat [16]. In the conventional programming process, SMPs are deformed at temperatures above their transition temperature (e.g., glass transition temperature), typically above room temperature, and then cooled below the transition temperature while holding the deformed shape, thereby securing a temporary configuration—a process known as hot programming [17, 18]. Although their responsive behavior is beneficial, the dependence on external heat for programming significantly limits their applicability in scenarios where heat exposure is inaccessible. Additionally, hot programming imposes a burden on SMPs when only localized morphing is required, as applying both precise heating and mechanical deformation complicates the programming process [19]. Remarkably, cold programming, a method of programming materials into a temporary form without requiring heat, presents a more flexible alternative, broadening the range of potential applications [17, 20]. As a non‐heating‐assisted method, it mainly relies on differential chemical or physical responses enabled by heterogeneous material systems [18, 21, 22]. For instance, heterogeneous hydrogel networks could exhibit cold‐programmable shape shifting in water due to their locally distinct swelling behaviors [21, 23, 24, 25, 26]. Nonetheless, the inherently slow water diffusion process and the dependency on aqueous conditions significantly limit the versatility and adaptability of these hydrogel systems [16]. Notably, force‐triggered shape‐memory systems, though less studied, offer a more attractive option for cold programmability [18, 22]. This mechanism mainly depends on the differential mechanical responses of the heterogeneous SMP system after being subjected to external force. Without the need for thermal or liquid conditions, these systems could achieve controlled shape‐morphing behavior. Notably, for successful implementation, it is crucial that one domain effectively stabilizes temporary shapes under ambient conditions after unloading, while the other domain demonstrates better mechanical resilience. Achieving these goals requires meticulous material design and the strategic construction of heterogeneous networks, a significant challenge that has been rarely addressed in existing research.

Furthermore, translating cold‐programmable shape‐memory function into bio‐derived systems poses greater material‐design challenges than in their petroleum‐derived counterparts. The intrinsic compositional and structural complexity of many bio‐based feedstocks often complicates the functionalization process and compromises the mechanical performance of the corresponding composites [27, 28, 29]. Overcoming these limitations requires innovative chemical modification strategies and/or precise microstructural control to ensure that properties such as shape‐memory actuation are retained or even enhanced [27, 30]. Nonetheless, achieving such multifunctional performance in bio‐derived systems is particularly rewarding, as it advances sustainability by reducing reliance on fossil‐based resources, lowering the carbon footprint, and promoting circular material lifecycles [31].

Building on their sustainability advantages, bio‐derived systems can be further advanced by drawing inspiration from nature, which provides remarkable design principles for the development of smart materials. Specifically, tendrils in climbing plants can transition from a straight to a coiled configuration in response to mechanical stimuli [32, 33]. This transformation arises from pronounced structural heterogeneity across the tendril cross‐section, which induces asymmetric deformation and enables rapid anchoring to external supports, thereby facilitating upward growth toward sunlight and other favorable niches [34, 35]. Such a natural strategy of structural differentiation and stimulus responsiveness serves as an invaluable blueprint for engineering synthetic materials with cold‐programmable shape‐memory capabilities [36].

Inspired by tendrils, whose cellular structural heterogeneity enables force‐responsive mechanical locking, we developed a cold‐programmable SMPC by deliberately engineering mesoscale morphological heterogeneity within a tailored glassy SMP matrix, on length scales intermediate between the molecular scale and the macroscopic sample dimensions. Sugar beet pulp (SBP), an abundant agricultural by‐product traditionally used as low‐value animal feed, was rationally processed and employed as a sustainable component to reduce reliance on fossil‐based resources [37]. The SBP was first ball‐milled into microparticles to alleviate its intrinsic structural complexity and subsequently magnetized with Fe_3_O_4_ nanoparticles, thereby converting the bio‐based feedstocks into functional components. Through a magnetically assisted curing strategy, the SMPCs were engineered with a heterogeneous architecture, in which the glassy SMP served as the shape‐stabilizing matrix while the SBP‐enriched domains acted as mesoscale stress regulators that facilitated local stress redistribution and spatially confined recovery. This bio‐inspired design enabled cold‐programmable actuation, a capability entirely absents in the homogeneous SMPC controls, which showed only negligible curvature changes upon uniaxial stretching. Importantly, unlike previously reported solvent‐mediated systems, temporary‐shape capture (cold programming) in our SMPCs was achieved through simple uniaxial stretching under ambient conditions, without any water or solvent involvement. This solvent‐independent mechanism eliminated diffusion‐governed delays and enabled sub‐minute programming, significantly expanding the operational domain of cold‐programmable materials. A side‐by‐side comparison (Table S1) highlights these distinctions relative to previously reported cold‐programmable systems. The potential of the cold‐programmable SMPCs was further demonstrated through deployable and protective structures for diverse applications such as aerospace systems. Overall, this work advances the functional scope of shape‐memory polymers by introducing a solvent‐independent cold‐programming mechanism while simultaneously enhancing sustainability through bio‐based material integration.

## 2 Results and Discussion

### 2.1 Bioinspired Mechanism

**FIGURE 1:**
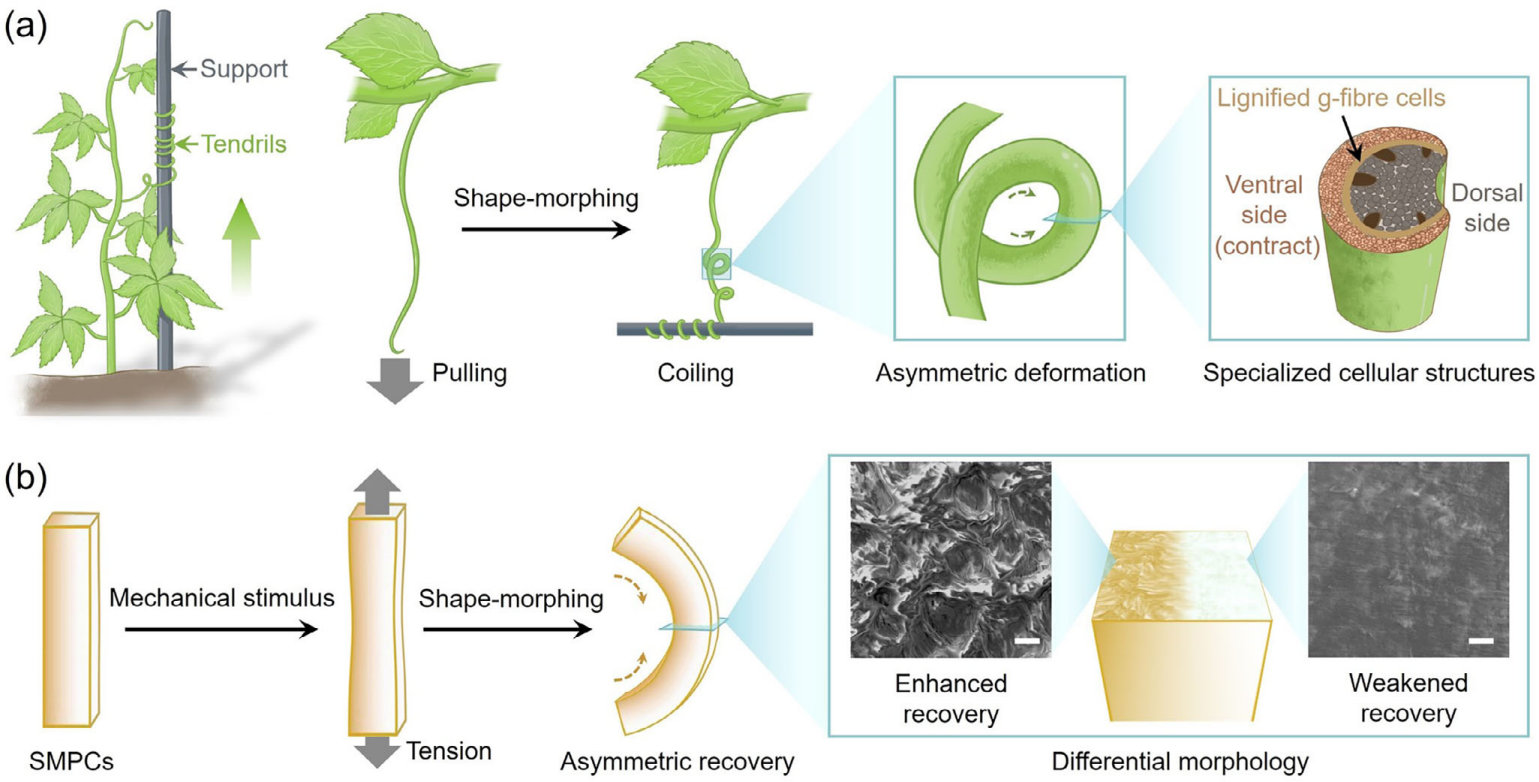
Vine tendril‐inspired polymer composites demonstrating cold‐programmable shape‐memory. (a) Illustration showing the morphing mechanism of a vine tendril, which coils around an object in response to a mechanical stimulus due to its unique cell structures. (b) Bioinspired SMPCs demonstrating shape‐morphing behaviors analogous to tendril systems, driven by differential morphology. Scale bars are 10 µm.

In nature, tendrils enable climbing plants to secure structural supports, promoting upward growth toward sunlight and access to beneficial ecological niches [34]. Upon encountering mechanical stimuli (e.g., pulling), the initially straight tendril undergoes a coiling transformation, anchoring itself to external supports (Figure 1a). This coiling behavior is enabled through a dynamic biomechanical process driven by the dorsiventral asymmetry in the tendril's specialized cellular structures. Notably, gelatinous fiber (g‐fiber) cells, which are morphologically differentiated and located along the ventral side of the curved tendril, undergo differential lignification in response to external forces [35, 38, 39]. This lignification induces longitudinal contraction on the ventral side relative to the dorsal side, resulting in macroscopic coiling of the tendril. Through this mechanism, tendrils exemplify force‐responsive, shape‐morphing capabilities.

Inspired by this natural response, we designed an SMPC system with SBP‐distribution‐induced asymmetric morphology. One textured region, enriched with SBP fillers, possesses enhanced resilience, enabling effective shape recovery after stretching. In contrast, the other region, mainly constructed by SMP networks, ensures the stabilization of temporary shapes following deformation. This heterogeneous design underpins the bioinspired deformation illustrated in Figure 1b. It should be noted that the heterogeneity in natural tendrils and that in the engineered SMPCs differ in origin: the former arises from asymmetric cellular organization, whereas the latter originates from the non‐uniform distribution of SBP fillers and the resulting mesoscale morphological heterogeneity. Nevertheless, both systems share the same functional principle, in which cross‐sectional heterogeneity generates differential mechanical responses and drives macroscopic shape morphing.

### 2.2 Design and Characterization of SMP Matrix

**FIGURE 2:**
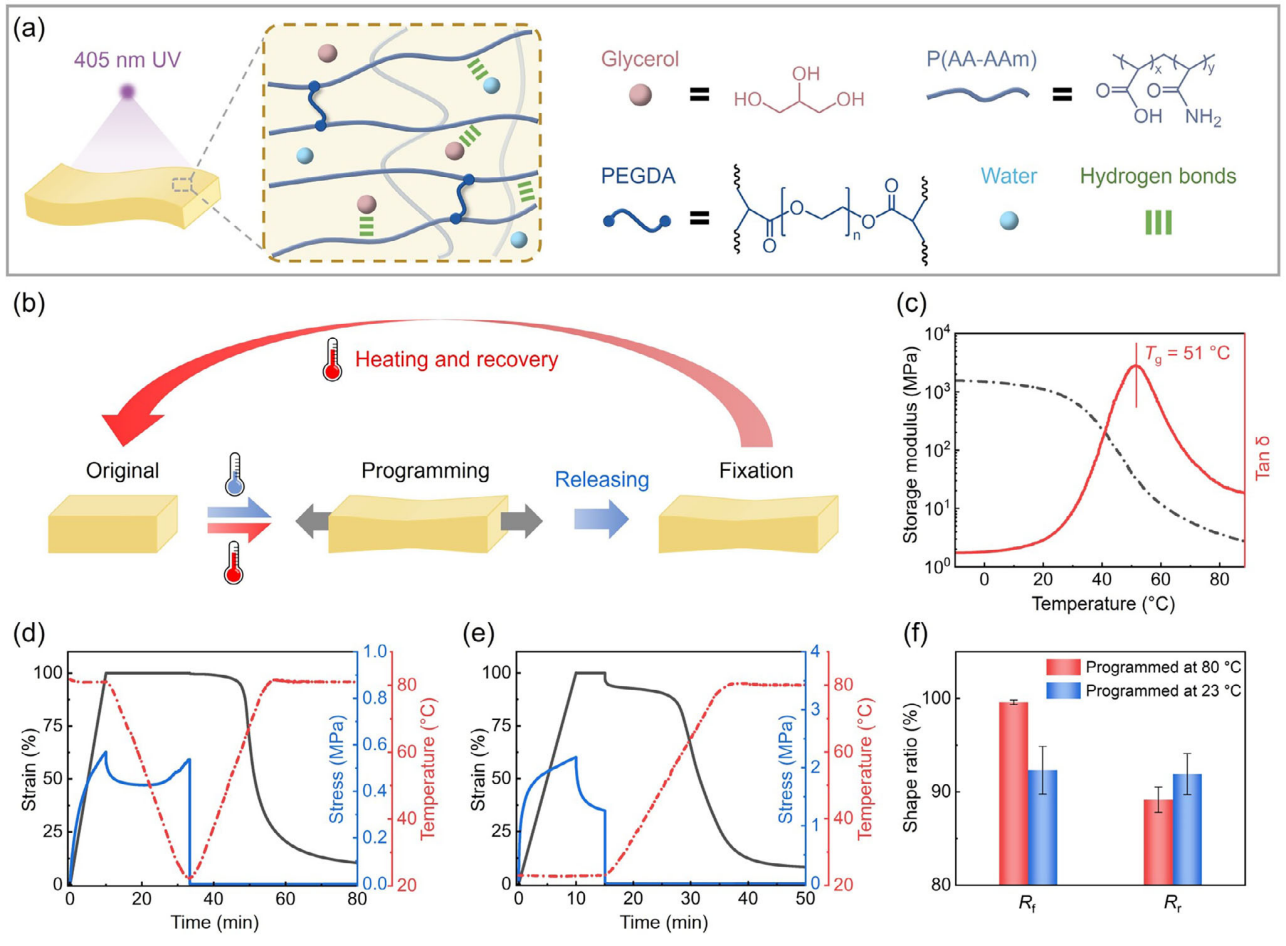
Details and characterizations of the formulated SMP matrix. (a) Network structures of UV‐cured SMPs. (b) Schematic illustration of the shape‐memory capabilities. (c) Storage modulus and tan δ as a function of temperature for the SMPs. (d) Shape‐memory behavior of the SMPs programmed at 80°C. (e) Shape‐memory behavior of the SMPs programmed at room temperature. (f) Summary of the shape‐fixing ratio (R_f_) and shape‐recovery ratio (R_r_) of the SMPs programmed at 80°C and at room temperature.

Prior to engineering heterogeneous SMPCs, it is essential to design the SMP network as a framework capable of stabilizing temporary shapes and facilitating effective shape recovery upon external heating. Figure 2a presents the chemical structure of the SMP, which was synthesized through photopolymerization under UV light. After photocuring, the resulting SMPs consist of poly(acrylic acid‐co‐acrylamide) (p(AA‐AAm), copolymerized from AA and AAm monomers) as the rigid polymer chain, poly(ethylene glycol) diacrylate (PEGDA) as the crosslinker, and glycerol and water as soft plasticizers. The proper balance between the stiff polymers and the soft plasticizers, which form hydrogen bonds, could achieve a suitable glass transition temperature (*T*_g_) for the SMP system, ensuring reliable shape fixation at ambient temperature [40, 41, 42]. Before evaluating the shape‐memory behaviors of the SMPs depicted in Figure 2b, we measured their *T*_g_ using dynamic mechanical analysis (DMA). As shown in Figure 2c, a *T*_g_ of 51°C was determined from the peak of the tan δ curve, indicating that the formulated SMPs could remain in a glassy state with restricted segmental mobility at room temperature (23°C) [40, 43, 44]. Consequently, deformation‐induced chain conformations could be immobilized within the network after unloading, enabling effective fixation of the temporary shape [45]. This process stored strain energy and locked the system into a non‐equilibrium configuration [22, 46]. Upon heating to 80°C, the polymer network entered a softened state with enhanced segmental mobility, enabling the release of stored energy and recovery of the permanent shape [15, 47].

Shape‐memory cycle tests were then performed on the SMPs using programming at 80°C (Figure 2d) and at room temperature (Figure 2e), respectively. In a typical hot‐programmed shape‐memory cycle, the SMPs were first elongated to 100% strain at a programming temperature of 80°C (i.e., *T*_g_ + 30°C). After cooling to room temperature, the shape‐fixing ratio (*R*_f_) of the SMPs was determined by releasing the external force. Shape recovery was then triggered by reheating, allowing the calculation of the shape‐recovery ratio (*R*_r_). As shown in Figure 2d and summarized in Figure 2f, the SMPs demonstrated effective shape‐memory behaviors, achieving shape‐fixing ratios approaching 100% and shape‐recovery ratios of approximately 90%. Multiple‐cycle shape‐memory tests were also conducted over five cycles to evaluate the cyclic stability of the SMPs. As shown in Figure S1, the SMPs maintained reliable shape‐memory performance within the tested cycling range.

The shape‐memory performance of the SMPs programmed at room temperature was also evaluated, as it is a critical prerequisite for enabling subsequent cold‐programmable shape morphing in heterogeneous SMPCs. It should be noted that the room‐temperature‐programmed shape‐memory behavior of the SMPs differs from the cold‐programmable actuation capability of the heterogeneous SMPCs. The former reflects the intrinsic shape‐fixing capability of the SMPs under uniaxial tensile deformation at room temperature and does not by itself generate curvature, whereas the latter arises from SBP‐induced mesoscale morphological heterogeneity, which enables force‐induced asymmetric recovery and controllable curvature generation, as discussed in Section 2.4.

In the room‐temperature‐programmed shape‐memory cycle, the SMPs were stretched to 100% strain at room temperature, and the shape‐fixing ratio was recorded upon unloading. Subsequently, the temperature was raised to 80°C to achieve shape recovery. As shown in Figure 2e,f, the SMPs exhibited high shape‐fixing and shape‐recovery ratios of approximately 92%. Notably, the shape‐fixing ratio of the SMPs after programming at room temperature was nearly 8% lower than that observed in the hot‐programmed tests. This discrepancy could be attributed to differences in chain relaxation behaviors under different deformation temperatures [8, 48]. Specifically, during programming at room temperature, chain relaxation was significantly restricted at room temperature, resulting in a higher driving force for shape recovery. Consequently, partial shape recovery occurred upon unloading, leading to a relatively lower shape fixity (Figure 2e,f). In contrast, during hot programming, stress relaxation was enhanced at elevated temperatures due to the accelerated exchange of dynamic hydrogen bonds [49]. This enhancement reduced the recovery force, allowing the shape to remain largely unchanged after unloading, as indicated by the relatively higher shape‐fixing ratio (Figure 2d,f). Collectively, the formulated SMP demonstrated the ability to stabilize temporary shapes and achieve reliable shape recovery upon external heating, irrespective of the programming temperature.

### 2.3 Mechanical Properties and Characterization of SMPs/SMPCs With Different SBP Contents

**FIGURE 3:**
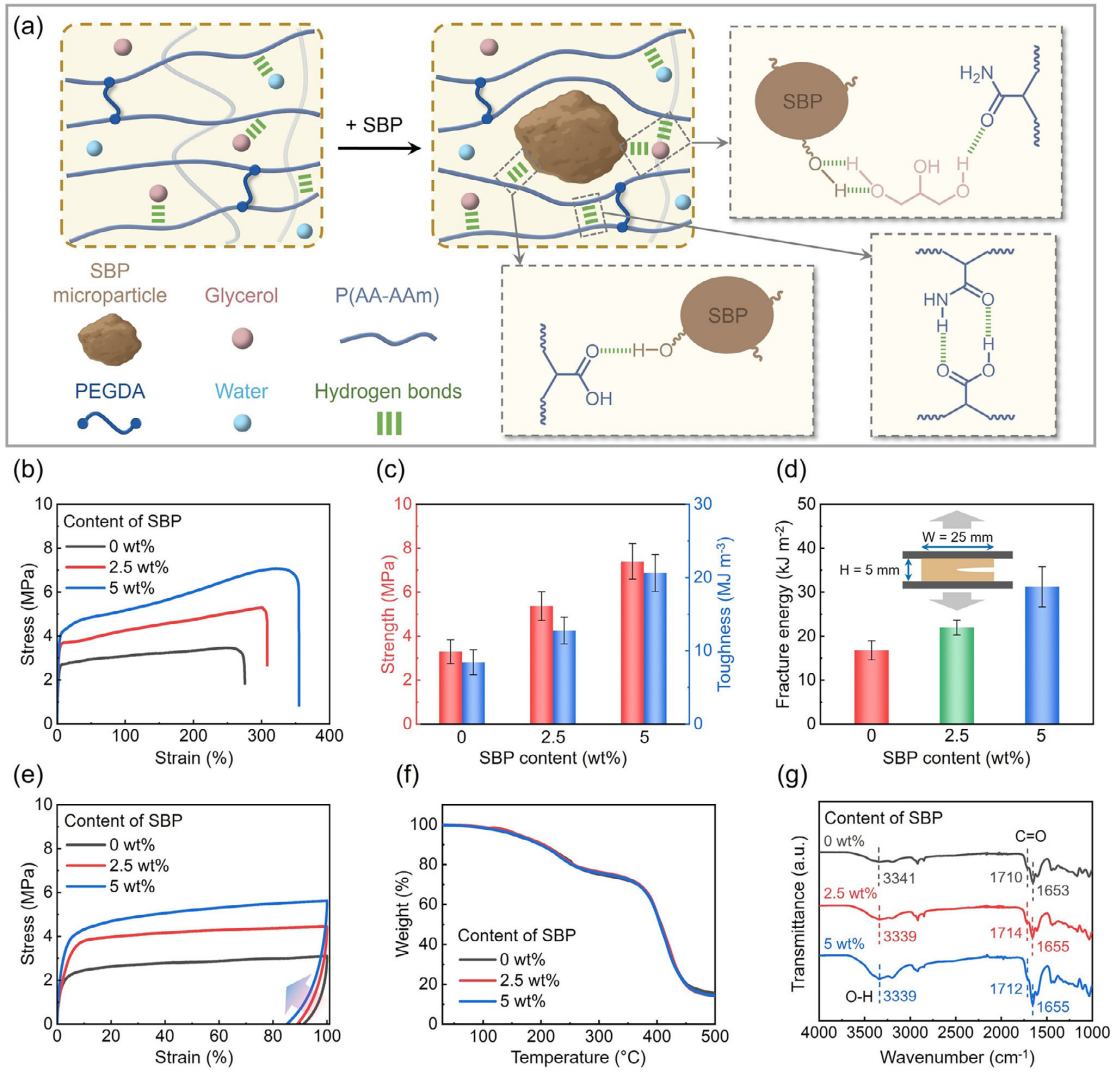
Mechanical properties and characterization of a homogeneous SMP system containing different SBP contents (0, 2.5, and 5 wt.%). (a) Structure comparison of the SMP system with and without SBP. (b) Tensile stress–strain behavior of SMP samples with different SBP contents. (c) Summary of tensile strength and toughness. (d) Fracture energy of the SMP system with different SBP contents. (e) Loading–unloading behavior of the SMP system with different SBP contents. (f) Thermal gravimetric analysis (TGA) of the SMP system with different SBP contents. (g) FTIR spectra of the SMPCs with different SBP contents.

To investigate the effect of SBP on the properties of the SMPCs, different contents of cleaned and milled SBP (0, 2.5, and 5 wt.%) were incorporated into the formulated SMP matrix to produce homogeneous SMPCs (Figure 3a). The samples were fabricated using a commercial bottom‐up DLP 3D printer (Elegoo Saturn 3 Ultra) equipped with a UV‐LED light projector. The rheological properties of resin ink are crucial for the DLP printing process, as the liquid resin must sufficiently refill the platform gap during each lift of the printing platform to enable the formation of successive layers [50]. To assess the suitability of the resins for DLP printing, the viscosity‐shear rate relationships were analyzed. As shown in Figure S2, the resins containing milled SBP demonstrated shear‐thinning, non‐Newtonian characteristics, with viscosity decreasing as the shear rate increased from 0.1 to 1000 s^−1^ [51]. The incorporation of milled SBP significantly increased the viscosity of the resin at low shear rates, likely due to the particle interactions [52]. However, as the shear rate increased, the viscosity decreased, which could be attributed to the alignment of suspended particles along the shear force direction [53, 54]. Notably, the resins exhibited relatively low viscosities (< 3 Pa·s) at elevated shear rates (30–1000 s^−1^), suggesting their suitability for DLP printing [55, 56]. Digital models for printing were then created using SolidWorks and sliced into 30 µm‐thick layers with CHITUBOX software. The SMPCs were printed layer by layer under UV light exposure, with each layer subjected to an exposure time of 30 s. Following the DLP printing process, the printed samples underwent post‐curing in a UV chamber (Form Cure, Formlabs) for 20 min to ensure complete polymerization and enhanced material performance.

Uniaxial tensile tests were conducted on printed dog‐bone‐shaped SMPC samples to characterize their mechanical properties. The resulting stress–strain curves are presented in Figure 3b, with the corresponding strength and toughness values summarized in Figure 3c. The results indicated that the introduction of SBP could improve the mechanical properties of the SMP system. Specifically, the mechanical strength increased from 3.3 MPa in neat SMP to 7.4 MPa in SMPCs containing 5 wt.% SBP, while toughness increased from 8.4 to 20.6 MJ m^−3^ under the same conditions. Additionally, pure shear tests were conducted on notched and unnotched specimens to measure the fracture energy of the SMP system with different SBP contents. As shown in Figure 3d, fracture energies increased with increasing SBP contents, demonstrating enhanced crack resistance attributed to SBP. Furthermore, loading–unloading tests were performed to compare the energy dissipation behavior of the SMPCs with different SBP contents, as presented in Figure 3e. The 5 wt.% SBP SMPCs exhibited the largest hysteresis loop, reflecting enhanced energy dissipation. Moreover, the unloading branch rose with increasing SBP content (Figure 3e), indicating a higher recovery stress. This trend suggests that SBP may not merely act as a bio‐based filler, but could also contribute to local stress redistribution within the glassy SMP matrix during deformation, thereby potentially introducing the mechanical asymmetry required for cold programming.

The thermal properties of the SMPCs were evaluated by thermal gravimetry. As shown in Figure 3f, the thermogravimetric analysis curves overlapped, which can be attributed to the negligible effects of SBP on thermostability. Furthermore, the influence of the SBP on the hot‐programmed shape‐memory properties was evaluated using homogeneous SMPCs containing 5 wt.% SBP. As shown in Figure S3, the homogeneous 5 wt.% SBP SMPCs retained effective shape fixation and thermally induced recovery, with a shape‐fixing ratio close to 100% and a shape‐recovery ratio of approximately 88%, both comparable to those of the neat SMPs. Dynamic mechanical analysis further showed that the tan δ peak shifted slightly from approximately 51°C for the neat SMPs to approximately 54°C for the homogeneous 5 wt.% SBP SMPCs (Figure S4). This slight increase in *T*_g_ suggested that SBP restricted the local segmental mobility of the SMP network, likely associated with hydrogen‐bonding interactions between the hydroxyl‐rich SBP and the SMP matrix. These results indicated that the incorporation of 5 wt.% SBP did not compromise the hot‐programmed shape‐memory function, while slightly altering the glass transition behavior of the SMP network.

Fourier‐transform infrared (FTIR) spectroscopy was then employed to verify the change in hydrogen bonding interactions induced by SBP incorporation. New hydrogen bonds could be formed between the carbonyl and hydroxyl groups of the SMP matrix and the abundant hydroxyl groups present in SBP (Figure 3a) [57, 58, 59, 60, 61]. As shown in Figure 3g, the absorption peak corresponding to the O─H group stretching shifted to a lower wavenumber (at 3339 cm^−1^) compared to that of the neat SMPs (at 3341 cm^−1^), indicating the formation of enhanced intermolecular hydrogen bonds (Figure 3a) [61, 62]. Additionally, the stretching peaks at 1710 and 1653 cm^−1^ corresponded to the C═O stretching of AA and AAm, respectively [63, 64]. Upon SBP incorporation, these C═O peaks shifted to higher wavenumbers (1712 and 1655 cm^−1^ for the 5 wt.% SBP sample), suggesting the formation of new hydrogen bonds between the carbonyl groups of the SMP matrix and the hydroxyl groups of SBP [57, 58, 59, 60].

### 2.4 Cold‐Programmable SMPCs Based on Differentially Distributed SBP

Heterogeneous SMPCs with spatially differentiated distributions of SBP were fabricated using a magnetically assisted photocuring process. It should be noted that the magnetized SBP content was fixed at 5 wt.% to balance formability and cold‐programmability of the SMPCs. Higher SBP contents were not adopted due to the UV‐shielding effect, where SBP hindered the photocuring and thus compromised the formability of the SMPCs.

**FIGURE 4:**
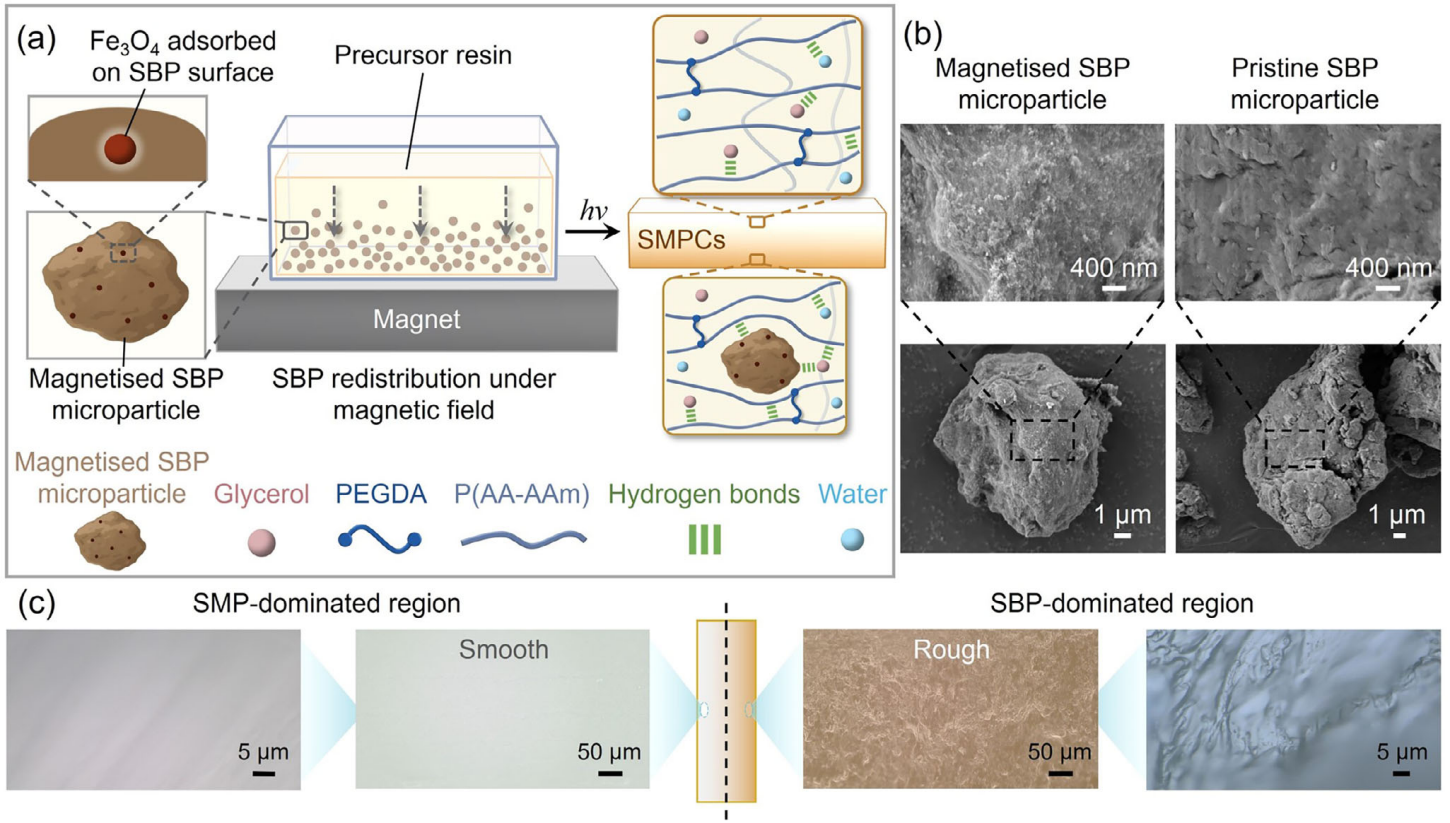
Preparation of the SMPCs with differentially distributed SBP. (a) Schematic illustration of the fabrication process. (b) SEM images comparing the morphology of magnetized SBP microparticles coated with Fe_3_O_4_ nanoparticles and pristine SBP microparticles. (c) Microscopic images of the SMPCs highlighting SMP‐dominated and SBP‐dominated regions.

As depicted in Figure 4a, milled SBP microparticles were first mixed with Fe_3_O_4_ nanoparticles, enabling the electrostatic adsorption of Fe_3_O_4_ onto the SBP surface. This adsorption was attributed to the anionic surfactant present on the iron oxide nanoparticles [65, 66]. Scanning electron microscopy (SEM) was employed to confirm the successful coating of iron oxide nanoparticles on SBP microparticles. As shown in Figure 4b, the magnetized SBP microparticle exhibited a distinctly granular texture compared to the unmodified SBP microparticle. Also, energy‐dispersive X‐ray spectroscopy (EDS) elemental mapping confirmed the presence of C, O, and Ca in the pristine SBP microparticles (Figure S5a), whereas the magnetized SBP showed an additional Fe signal (Figure S5b), thereby confirming the successful coating of Fe_3_O_4_ nanoparticles onto the SBP surface.

The effective adsorption of Fe_3_O_4_ was further validated by the appearance of a clear supernatant following magnetic‐assisted sedimentation of the magnetized SBP (Figure S6). Initially, the mixture of Fe_3_O_4_ ferrofluid and SBP suspension appeared as a homogeneous dark‐brown dispersion (Figure S6a), since Fe_3_O_4_ nanoparticles (dark‐brown) were uniformly suspended in water and had not yet been adsorbed by SBP. After 24 h of agitation, the Fe_3_O_4_ nanoparticles were effectively immobilized on the SBP through electrostatic attraction. Upon magnetic‐assisted sedimentation, the magnetized SBP‐Fe_3_O_4_ composites were rapidly attracted toward the bottom, thus leaving a clear supernatant (Figure S6b). Such a distinct separation occurs only when Fe_3_O_4_ is firmly anchored to the SBP rather than freely dispersed in the liquid [66]. The magnetization of the SBP filler was conclusively demonstrated by its attraction to a magnet, as presented in Figure S7 and Movie S1.

Subsequently, the magnetized SBP was freeze‐dried and incorporated into the resin precursor. This mixture was deposited into a transparent mold placed in direct contact with the surface of the neodymium magnet, as illustrated in Figure 4a. The system was then left undisturbed for 30 min to allow the magnetized SBP to migrate and stabilize within the resin precursor. The mold was subsequently transferred to a UV chamber (Form Cure, Formlabs) for an initial photocuring for 20 min. Following mold removal, an additional photocuring step lasting 30 min was performed to ensure complete polymerization of the SMPC networks. Cross‐sectional microscopic observations and visual color contrast indicated that the magnetized SBP particles were mainly localized near the magnet‐facing side rather than being linearly distributed throughout the SMPC thickness. Under the fixed fabrication conditions used in this study, including 5 wt.% magnetized SBP, fixed magnet configuration, and 30 min stabilization time, an SBP‐rich layer with an estimated thickness of approximately 0.8 mm was formed and subsequently fixed during photocuring.

The resultant SMPCs exhibited a heterogeneous internal morphology, as illustrated in Figure 4a. A digital microscope and a profilometer were employed to characterize the morphological differences. As shown in Figure 4c and Table S2, the region dominated by the SMP matrix presented a relatively smooth surface with a low roughness (*Ra* = 0.0834 µm). By contrast, the SBP‐rich region exhibited a markedly rougher microstructure with branched features, as evidenced by a significantly higher *Ra* of 0.3995 µm. The incorporation of SBP thus disrupted the continuity of the polymer matrix and introduced a distinct mesoscale architecture absent in the SMP region. Such differences could contribute to the divergent stress responses observed across SBP‐loaded systems during loading–unloading (Figure 3e).

**FIGURE 5:**
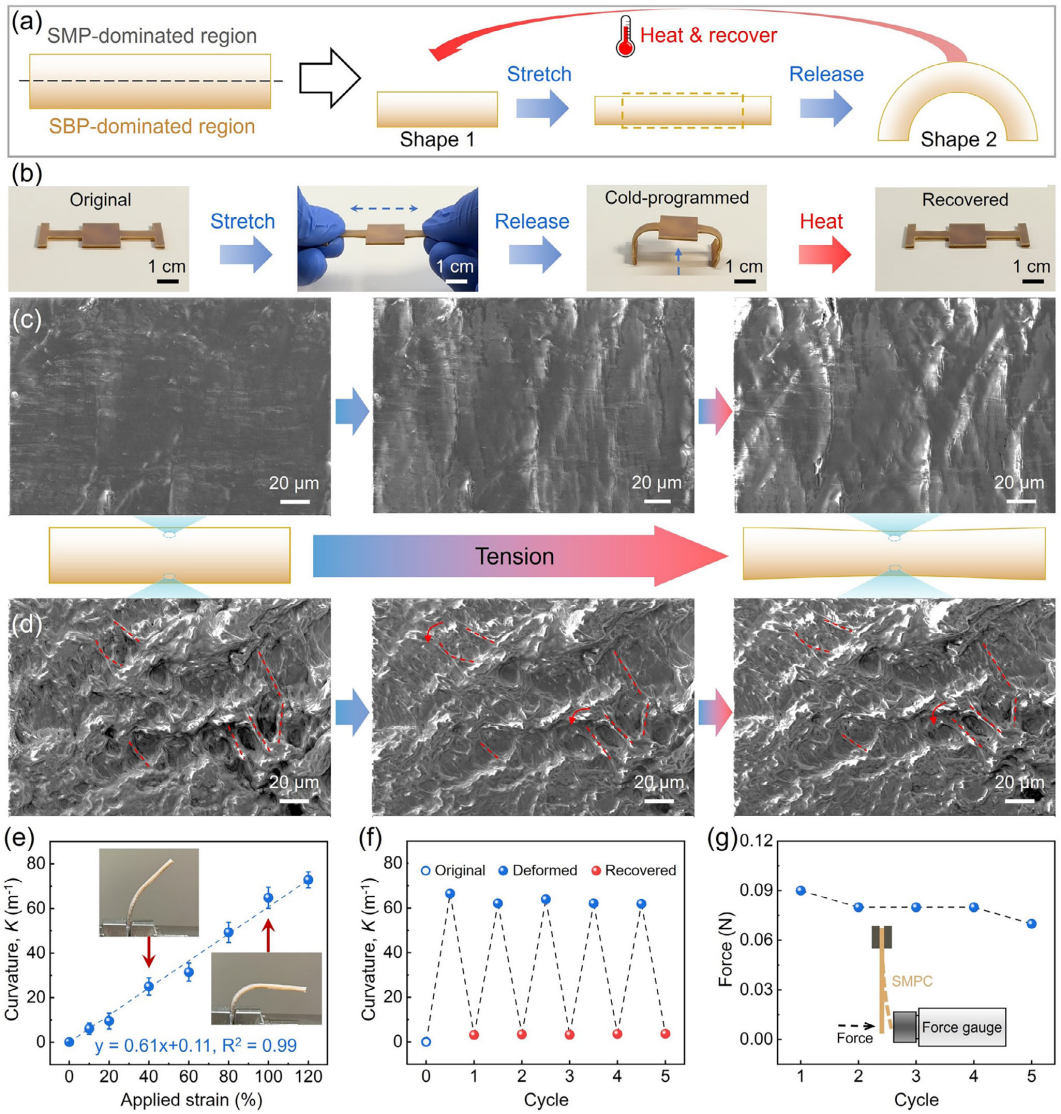
Cold‐programmable shape‐memory behavior of the SMPCs with differentially distributed SBP (total SBP content: 5 wt.%). (a) Schematic illustration of the cold‐programmable shape‐memory capabilities. (b) Photographs illustrating the cold‐programmed shape‐memory response of the SMPC‐based robot. (c) In situ SEM images of the SMPCs under tensile loading in the SMP‐dominated region. (d) In situ SEM images of the SMPCs under tensile loading in the SBP‐dominated region. (e) Curvature as a function of applied strain for SMPCs (sample dimensions: 70 mm × 6 mm × 3 mm). (f) Curvature reversibility over multiple response cycles (five cycles) under 100% applied strain (sample dimensions: 70 mm × 6 mm × 3 mm). (g) Actuation force reversibility over multiple response cycles (five cycles) under 100% applied strain (actuation region dimensions: 50 mm × 6 mm × 3 mm; initial gap of 5 mm between the SMPC strip and the force gauge).

By employing the magnetically assisted photocuring approach, the SMPCs were endowed with cold‐programmable shape‐memory capabilities. Notably, unlike previously reported magnetic‐field‐responsive SMPC systems that primarily employed magnetic fillers for magnetothermal heating and/or magnetic‐field‐driven actuation [67, 68, 69], the magnetic field in this work was applied only during fabrication to spatially redistribute magnetized SBP and construct mesoscale morphological heterogeneity. The subsequent programming and shape morphing were achieved by simple uniaxial stretching under ambient conditions, without magnetic‐field actuation, heating, or solvent assistance during programming. As shown in Figure 5a,b and Movie S2, the resulting SMPCs could be cold‐programmed into a temporary shape via stretching and subsequently recover their original shape upon heating. This functionality arose from the asymmetric mechanical responses exhibited by different regions of the SMPCs. Specifically, the region dominated by the SMP matrix was capable of stabilizing a temporary shape at room temperature (Figure 2e), accompanied by relatively low unloading stress (Figure 3e), which restricted substantial shape recovery after stretching. In contrast, the region enriched with SBP particles showed relatively higher unloading stress (Figure 3e), promoting enhanced elastic recovery at room temperature. Upon heating, the entire system underwent softening of the SMP network, enabling recovery from plastic deformation. Consequently, cold‐programmable shape‐memory capability was achieved.

To further elucidate the mechanisms of the asymmetric mechanical responses in the heterogeneous SMPCs, we performed in situ SEM observations under uniaxial tensile loading. As shown in Figure 5c,d, the two regions exhibited distinct deformation modes. In the SMP‐dominated region (Figure 5c), the initially smooth surface progressively developed pronounced plasticity‐induced shear bands with increasing strain [70]. This evolution indicated the restricted molecular mobility of the chain segments in the glassy state, which limited elastic recovery and thereby stabilized the temporary shape [22, 70, 71, 72]. By contrast, the SBP‐enriched region exhibited branched structures that underwent ductile torsion and stretching under load, as highlighted by the red arrows in Figure 5d. Such morphological features could alleviate localized stress concentrations and improve shape recovery [73, 74]. This behavior further demonstrated that SBP not only altered the mesoscale morphology but also actively regulated stress redistribution during deformation. As a result, the resulting morphological heterogeneity across the SMPC cross‐section produced a built‐in mechanical asymmetry between the two domains, which is essential for enabling cold programming.

To characterize the actuation behavior of the SMPCs as a function of the applied strain, we conducted tests involving the stretching and releasing of SMPC samples (with dimensions of 70 mm × 6 mm × 3 mm), as depicted in Figure S8. Notably, the morphing behavior could be more accurately described using the dimensionless parameter of curvature, rather than the bending angle, as curvature is independent of the gauge length of the samples. The results, presented in Figure 5e, revealed that the curvature increased progressively with the applied strain, highlighting the controllable actuation performance of the heterogeneous SMPCs. Moreover, the shape‐morphing behavior of the SMPCs was shown to be repeatable over multiple cycles, with small variations in curvature (Figure 5f; Figure S9). Although the actuation force exhibited a moderate decrease during the initial cycles, it subsequently reached a relatively stable level (Figure 5g; Figure S10). This initial force attenuation is likely associated with partial stress relaxation of the heterogeneous SMPC network during repeated deformation‐recovery cycles.

Overall, the magnetically assisted photocuring strategy endowed the SMPCs with cold‐programmable actuation that was completely absent in the compositionally uniform controls. As shown in Figure S11, homogeneous SMPCs generated negligible curvature changes upon uniaxial stretching and therefore failed to exhibit cold‐programmable actuation. In contrast, the magnetically induced heterogeneous architecture introduced a resilient SBP‐dominated domain within a robust SMP matrix, establishing the mechanical asymmetry required for actuation. This synergistic configuration facilitated cold‐programmable SMPCs with tunable and precise actuation performance, drawing inspiration from the stimuli‐responsive, structurally mediated shape transformations observed in vine tendrils.

### 2.5 Showcase Applications of Cold‐Programmable SMPCs

**FIGURE 6:**
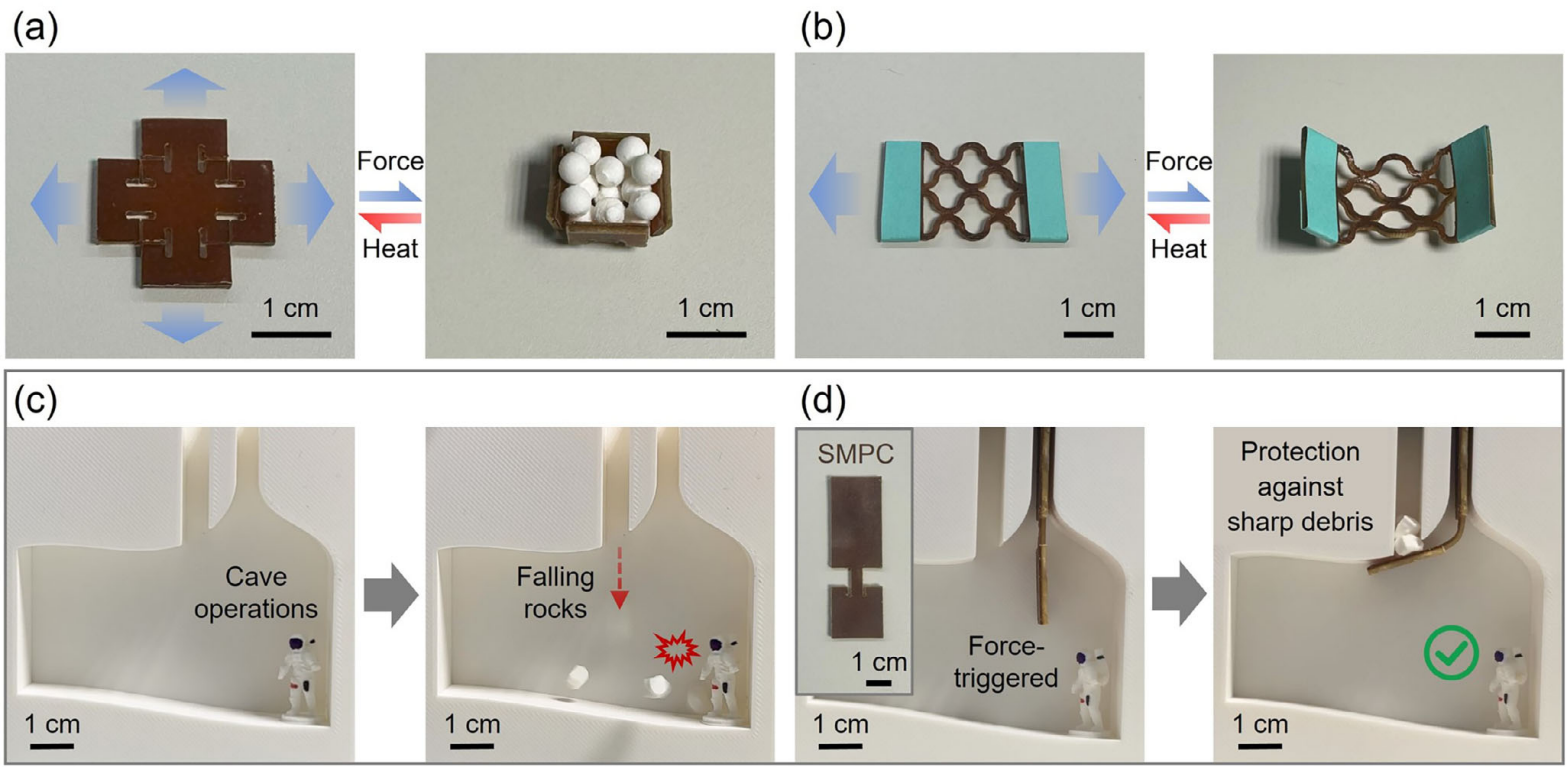
Application demonstrations of cold‐programmable SMPCs for deployable and protective structures. (a) Photographs of a compact container made of cold‐programmable SMPCs, exhibiting mechanically induced folding. (b) Photographs of a deployable grid structure made of cold‐programmable SMPCs, activated via uniaxial stretching. (c) Illustration of a rockfall incident during cave operations in an aerospace setting. (d) Illustration of the deployment of an SMPC‐based protective gate, activated through accessible mechanical inputs to prevent sharp debris from further descent. The inset shows the front view of the undeformed SMPC before activation.

To demonstrate the prospective utilization of cold‐programmable SMPCs, we designed a series of deployable structures that showcase their distinct advantages over conventional shape‐memory materials, which typically rely on thermal programming for shape fixation. Figure 6a presents a compact SMPC sheet that could fold into a container upon feasible stretching. This capability not only enabled heat‐free actuation but also offered a space‐saving solution for applications such as packaging, logistics, and field‐deployable equipment, where volume efficiency and ease of deployment are essential [75, 76, 77]. In another example, we fabricated a grid‐patterned SMPC structure that transformed from a planar to a concave, shell‐like configuration upon uniaxial stretching (Figure 6b). This mechanically induced reconfiguration is particularly valuable for applications in soft robotics, energy storage systems, and biomedical devices, where geometric adaptability under mild stimuli is desirable [78, 79, 80].

Leveraging the cold‐programmable feature of the SMPCs, we further demonstrated their use as a protective device in an aerospace setting (Figure 6c,d). In such scenarios, power availability is often severely constrained, and the complexity and mass of conventional mechanical systems pose significant challenges for transportation and on‐site deployment [81, 82, 83, 84, 85]. Meanwhile, the advancement of space exploration technologies has prompted new demands for extraterrestrial geological operations, including subsurface excavation in lunar or Martian caves [86, 87]. These environments present inherent geotechnical hazards, such as rockfalls (Figure 6c; Movie S3), posing significant risks to astronauts during in situ exploration, habitat construction, and resource harvesting missions.

To mitigate these risks, we developed SMPC‐based protective structures that could be deployed through accessible mechanical inputs (e.g., an astronaut's body weight or operational tools) to form effective barriers around potential rockfall sources, thereby preventing sharp debris from further descent (Figure 6d; Movie S3). Unlike conventional SMP systems, this design operated without an onboard power supply and circumvented the impracticality of in situ thermal programming in resource‐limited scenarios such as aerospace. It should be noted that this demonstration served as a proof‐of‐concept, highlighting the feasibility of constructing reconfigurable protective structures based on our cold‐programmable SMPC system, rather than representing a full‐strength device. As shown in Figure S12, the load‐bearing capability of the protective SMPC samples was quantitatively evaluated, revealing moderate performance primarily limited by the fabrication parameters (e.g., magnet size and UV‐curing conditions). In practical applications, the structural geometry and programming load could be further customized to mission‐specific requirements to enhance mechanical robustness and protective performance.

## 3 Conclusions

In this work, we developed a bio‐derived SMPC system with SBP‐distribution‐induced heterogeneous configurations to achieve cold‐programmable shape‐memory functionality. Sugar beet pulp, an abundant low‐value agricultural by‐product, was strategically modified and integrated as a bio‐based component to enhance mechanical robustness while reducing dependence on fossil‐based resources. Inspired by vine tendrils, whose asymmetric cellular structures enable force‐responsive coiling, the SMPCs were engineered via a magnetically assisted curing process to establish distinct functional domains: a formulated SMP matrix that provided structural stability, and the SBP‐enriched domains with rough morphologies that imparted enhanced resilience. This bio‐inspired and bio‐derived integration endowed the SMPCs with force‐responsive actuation, driven by differential resilience after deformation, thereby achieving cold‐programmable shape‐memory behavior without the need for thermal or aqueous stimuli. The resulting morphing responses were repeatable across multiple cycles and could be precisely controlled by applied strain. Finally, the applicability of the cold‐programmable SMPCs was demonstrated through deployable and protective structures, underscoring their potential in demanding scenarios such as aerospace systems. Overall, this work introduces a novel cold‐programmable shape‐memory concept and highlights the transformative role of bio‐based materials in advancing sustainable smart material design.

## 4 Experimental Section

### 4.1 Materials

Glycerol (≥ 99.5%), acrylic acid (AA), acrylamide (AAm, ≥ 98%), Poly(ethylene glycol) diacrylate (PEGDA, average *M*_n_ = 575 g mol^−1^), and phenylbis(2,4,6‐trimethylbenzoyl) phosphine oxide (Irgacure 819, 97%) were bought from Sigma–Aldrich and used without any purification. Ferrofluid EMG 705 was supplied by Ferrotech (Bedford, USA) and used without further purification. The neodymium magnet (N52 grade, surface magnetic field ∼343 mT; dimensions: 85 mm × 34 mm × 20 mm) was purchased from Fuyuan (Shenzhen, China). Sugar beet pulp (SBP) was received from AB Sugar (Peterborough, UK).

### 4.2 Cleaning of SBP

A total of 500 g (dry weight) of as‐received SBP was added to 500 mL of 0.5 M HCl in a 2 L Erlenmeyer flask. The mixture was treated in an autoclave (Astell AMB Classic Benchtop Autoclave) under saturated steam at 15 psi for 30 min. After cooling to room temperature, the mixture was neutralized with 2 M NaOH. The SBP was then washed twice with ultrapure water and dried in an oven (Memmert Universal Oven UF55) at 50°C for 24 h.

### 4.3 Magnetization of SBP

Before magnetization, the cleaned SBP was milled using a planetary ball‐milling machine (Retsch, PM 100). The SBP and zirconia milling balls (10 mm in diameter) were loaded into the milling jar at a mass ratio of 1:10, filling about half of the jar volume. Milling was conducted at 500 rpm for 3 h. The morphology of the resulting SBP particles is shown in Figure S13. After that, 6 g of milled SBP was dispersed in 180 mL of deionized water and stirred at room temperature. 225 µL of ferrofluid EMG 705 was then diluted in 120 mL of deionized water and added to the above SBP suspension. EMG 705 contains 3.9 vol% magnetite (Fe_3_O_4_) particles (Ferrotech, USA). Thus, the nominal volume of magnetite particles present in the added ferrofluid was approximately 8.8 µL, corresponding to a nominal magnetite/SBP volume‐to‐mass ratio of approximately 1.5 µL g^−1^. The Fe_3_O_4_ particles in the ferrofluid EMG 705, approximately 12 nm in size, were electrostatically adsorbed onto the SBP surface due to the presence of anionic surfactant on the iron oxide nanoparticles [65, 66]. To ensure their successful adsorption, the resulting mixture was continuously stirred for 24 h at room temperature. The magnetized SBP was then obtained through a freeze–drying process, utilizing a freeze dryer (Labconco FreeZone 4.5 L).

### 4.4 Preparation of Precursor Resins

To prepare the SBP‐free SMP resins, the monomers (AA and AAm) and crosslinker (PEGDA) were mixed with glycerol and deionized water in a light‐shielded glass bottle. Subsequently, the photoinitiator Irgacure 819 (2 wt.% relative to the combined mass of the monomers and PEGDA) was added, and the mixture was stirred at 50°C for 30 min to obtain a homogeneous SMP solution. To prepare the SBP‐containing precursor for the homogeneous SMP/SMPC samples, different contents of milled SBP (processed via ball milling using a Retsch PM 100 machine at 500 rpm for 3 h) were introduced into the above SMP solution. The resulting mixture was agitated at room temperature for 30 min to achieve uniform dispersion. For the precursor containing magnetized SBP, different contents of magnetized SBP were mixed with the SMP solution, followed by agitation at room temperature for 30 min. Detailed compositions of the precursor resins are listed in Table S3.

### 4.5 Fabrication of Homogeneous SMP/SMPC Samples

The homogeneous samples were prepared using a commercial bottom‐up DLP 3D printer equipped with 405 nm UV light. All samples were printed under identical printing conditions to ensure a consistent curing degree across the series. The 3D models were designed in SolidWorks and sliced into 30 µm‐thick layers using CHITUBOX. The precursor resin, with or without milled SBP, was poured into the printer tank, and printing was carried out layer by layer under UV light irradiation (30–40 s exposure time for each layer). After printing, the samples underwent photocuring in a 405 nm‐UV chamber (Form Cure, Formlabs) for 30 min.

### 4.6 Fabrication of SMPCs with Differentially Distributed SBP

The SMPCs with differentially distributed SBP were fabricated via a magnetically assisted photocuring process. First, the precursor resin containing magnetized SBP was dispensed into a transparent mold placed in direct contact with the surface of a neodymium magnet (N52, Fuyuan), as illustrated in Figure 4a. The entire setup was kept undisturbed for 30 min, allowing the magnetized SBP to migrate and subsequently stabilize within the resin matrix. Subsequently, the mold was carefully transferred to a UV curing chamber (Form Cure, Formlabs) for an initial exposure of 20 min. After demolding, an additional photocuring lasting 30 min was performed to ensure complete polymerization of the SMPCs. To obtain the patterned samples, the cured SMPCs were further laser‐cut using a VLS6.60 laser platform (Universal Laser Systems).

### 4.7 Design and Fabrication of the Cave‐Shaped Model

The cave‐shaped model was fabricated using a fused filament fabrication (FFF) 3D printer (Ultimaker 3) with commercial polylactic acid (PLA) filaments (2.85 mm diameter, Ultimaker). The geometric design was first developed using SolidWorks, and the resulting model was subsequently sliced with Ultimaker Cura software before FFF 3D printing.

### 4.8 Characterization

Fourier transform infrared (FTIR) spectra of the SMPs were recorded using a Spectrum Two FTIR spectrometer (PerkinElmer, ATR mode) in the region of 4000–600 cm^−1^. Thermal gravimetric analysis (TGA) was conducted with an SDT 650 system (TA Instruments) from 23°C to 500°C in a nitrogen atmosphere at a heating rate of 10°C min^−1^. The iron oxide nanoparticles coated on SBP were visualized using a Zeiss Gemini 360 scanning electron microscope (SEM). Samples were mounted on metal stubs with double‐sided adhesive carbon tape and observed at an accelerating voltage of 3 kV. The morphology of the milled SBP particles was characterized by scanning electron microscopy (Zeiss Gemini 360) operated at an accelerating voltage of 5 kV. In situ SEM measurements of the SMPCs were conducted using a Zeiss Gemini 360 SEM with a Deben Microtest tensile stage (2 kN load cell). Samples were clamped and observed at an accelerating voltage of 3 kV. Morphologies of the SMPCs in different regions were observed using a digital microscope (Keyence, VHX‐7000). The surface roughness of the SMPCs in different regions was measured through an optical profilometer (Alicona InfiniteFocus G6). Measurements were conducted over an area of 777 µm × 777 µm with a lateral sampling interval of 0.36 µm. The rheological properties of the precursor resins prior to 3D printing were evaluated using a Discovery Hybrid Rheometer (DHR‐3, TA Instruments). Viscosities were measured across shear rates from 0.1 to 1000 s^−1^ at room temperature (23°C). Dynamic mechanical analysis (DMA) was performed using a Q850 DMA apparatus (TA Instruments) in tension mode, with rectangular samples (20 mm × 3 mm × 2 mm) tested at a heating rate of 3°C min^−1^ from −10°C to 90°C, at a frequency of 1 Hz and 0.1% strain amplitude. The glass transition temperature (*T*_g_) was determined as the temperature corresponding to the peak in the tan δ curve. To characterize the actuation behavior of the SMPCs as a function of the applied strain, their deformation was captured using a commercial mobile phone, and the resulting curvature was quantified with ImageJ software.

### 4.9 Mechanical Measurements

Uniaxial tensile tests were conducted on dog‐bone‐shaped samples (ASTM D638) using an Instron 5969 machine equipped with a 10 kN load cell at a stretching rate of 10 mm min^−1^. For the loading–unloading tests, specimens were stretched to 100% strain and then unloaded to zero force at the same rate to complete each cycle. Pure shear tests were conducted on both notched and unnotched specimens to measure the fracture energy of the SMP system at different SBP concentrations. The sample dimensions for pure shear tests were 50 mm × 25 mm × 2 mm, with notched specimens featuring a notch length of approximately half the sample width (∼12.5 mm). The force–displacement curves for unnotched samples were recorded as shown in Figure S14, and the corresponding notched samples were stretched to determine the critical displacement (*λ*_c_) at the rupture. Fracture energy (*Γ*) was then calculated by integrating the force–displacement curve of the unnotched sample at *λ*_c_ as follows:

(1) $$\Gamma =\frac{W\left(\lambda_{c}\right)}{A}$$

where *W*(*λ*_c_) represents the work to stretch the unnotched sample to *λ*_c_, and *A* is the cross‐sectional area of the unnotched sample.

### 4.10 Shape‐Memory Tests

The shape‐memory behaviors were evaluated using a Q850 DMA apparatus (TA Instruments). For the hot‐programmed shape‐memory tests, rectangular SMP samples (20 mm × 3 mm × 2 mm) were first stretched to 100% strain (*ε*_p_) at a programming temperature of 80°C. The temperature was then gradually reduced to room temperature (23°C) while maintaining the 100% strain. Once cooled, the tensile load was removed, allowing the fixed strain (*ε*_u_) to be obtained, and the shape‐fixing ratio (*R*_f_ = *ε*_u_/*ε*_p_) was calculated. The samples were then reheated to the programming temperature (80°C) to enable the free recovery of the deformed shape, and the shape‐recovery ratio (*R*_r_ = (*ε*_u_−*ε*_r_)/*ε*_u_) was determined using the recovery strain (*ε*_r_). Room‐temperature‐programmed shape‐memory tests followed a similar procedure: samples were stretched to 100% strain at room temperature (23°C) to achieve a temporary shape. The temperature was then increased without applying a tensile load, allowing for shape recovery.

## Conflicts of Interest

The authors declare no conflicts of interest.

## Supporting information




**Supporting File 1**: smll74830‐sup‐0001‐SuppMat.docx.

**Supporting File 2**: smll74830‐sup‐0002‐MovieS1.mp4.

**Supporting File 3**: smll74830‐sup‐0003‐MovieS2.mp4.

**Supporting File 4**: smll74830‐sup‐0004‐MovieS3.mp4.

## Data Availability

The data that support the findings of this study are available from the corresponding author upon reasonable request.
