# Supplementary material for: Vine Tendril‐Inspired Cold‐Programmable Shape‐Memory Polymer Composites Using Sugar Beet Pulp
Source: Small. 2026 Jul 29;22(54):e74830. doi: 10.1002/smll.74830 (PMC13614295; doi:10.1002/smll.74830)
Supplement: Supplementary file 1 [file SMLL-22-e74830-s003.docx]

Supporting Information

**Vine Tendril-Inspired Cold-Programmable Shape-Memory Polymer Composites Using Sugar Beet Pulp**

*Wei Huang, Vikramjeet Singh, Yu Wang, Junhao Chen, Mingjing Zhang, Gary J. Lye, Helen C. Hailes,* *Luna Jiang, Eral Bele, Manish K. Tiwari**

Dr. W. Huang, J. Chen, Prof. M. K. Tiwari

Nanoengineered Systems Laboratory, UCL Mechanical Engineering, University College London, London, WC1E 7JE, UK.

E-mail: [m.tiwari@ucl.ac.uk](mailto:m.tiwari@ucl.ac.uk)

Dr. W. Huang, J. Chen, Prof. M. K. Tiwari

UCL Hawkes Institute, University College London, London, W1W 7TS, UK.

Dr. V. Singh, Dr. E. Bele, Prof. M. K. Tiwari

Manufacturing Futures Laboratory, University College London, London, E20 2AE, UK.

Dr. Y. Wang, Prof. G. J. Lye

Department of Biochemical Engineering, University College London, London, WC1H 0AH, UK.

Dr. Y. Wang, Prof. H. C. Hailes

Department of Chemistry, University College London, London, WC1H 0AJ, UK.

M. Zhang

Institute of Orthopaedic and Musculoskeletal Science, University College London, Royal National Orthopaedic Hospital, London, HA7 4LP, UK.

Dr. L. Jiang

Advanced Characterisation Lab for Materials and Manufacturing, UCL Mechanical Engineering, University College London, London, WC1E 7JE, UK.

Keywords: bio-inspired strategies, bio-derived materials, shape-memory polymers, cold programming, sugar beet pulp

**This file includes:**

Figures S1 to S14

Tables S1 to S3

**Other Supplementary Material for this manuscript includes the following:**

Movies S1 to S3

**Figure S1.** Cyclic shape-memory performance of the SMPs over five cycles.

**Figure S2.** Viscosity-shear rate curves of the precursor resins with different SBP contents (0 wt%, 2.5 wt%, and 5 wt%).

**Figure S3.** Shape-recovery behaviour of the neat SMPs and homogeneous SMPCs containing 5 wt% SBP.

**Figure S4.** Tan δ curves of the neat SMPs and homogeneous SMPCs containing 5 wt% SBP.

**Figure S5.** Scanning electron microscopy (SEM) images and energy-dispersive X-ray spectroscopy (EDS) mapping of (a) pristine SBP microparticle and (b) magnetised SBP microparticle coated with Fe_3_O_4_ nanoparticles.

**Figure S6.** Photographs of the mixture of Fe_3_O_4_ and SBP suspension (a) before and (b) after the adsorption of Fe_3_O_4_ nanoparticles onto SBP.

**Figure S7.** Magnetic attraction behaviour of the SBP coated with Fe_3_O_4_ nanoparticles.

**Figure S8.** Schematic illustration of the actuation tests.

**Figure S9.** Curvature reversibility over twenty response cycles under 100% applied strain (sample dimensions: 70 mm × 6 mm × 3 mm).

**Figure S10.** Actuation force reversibility over twenty response cycles under 100% applied strain (sample dimensions: 70 mm × 6 mm × 3 mm).

**Figure S11.** Curvature response of homogeneous SMPCs containing 5 wt% SBP under 100% applied strain (sample dimensions: 70 mm × 6 mm × 3 mm).

**Figure S12.** Load-bearing capacity of protective SMPC samples as a function of actuation region width and sample thickness.

**Figure S13.** Scanning electron microscopy (SEM) characterisation of SBP microparticles after ball milling treatment. The scale bar is 10 µm.

**Figure S14.** Force-displacement curves for unnotched and notched samples with SBP contents of (a) 0 wt%, (b) 2.5 wt%, and (c) 5 wt%.

**Table S1.** A comparison between our work and state-of-the-art shape-memory systems.

| Materials | Sustainable source | Programming  type | Response  time | Shape fixity | Shape recovery | Reference |
| --- | --- | --- | --- | --- | --- | --- |
| Poly(*tert*-butyl acrylate-co- isobornyl acrylate) (P(*t*BA-IBOA))/aliphatic urethane diacrylate (AUD) | None | Hot programming at 80 ºC | - | > 95% | > 80% | [1] |
| Poly(isobornyl acrylate) (PIBOA)/poly(ethylene glycol) diacrylate (PEGDA)/tricyclo[5.2.1.0^2,6^] decanedimethanol diacrylate (TcddA) | None | Hot programming at 80 ºC | - | 98% | 97% | [2] |
| Poly(acrylic acid) (PAA)/*N*,*N*’-methylenebisacrylamide (MBAA)/calcium acetate | None | Hot programming in 90 °C water | > 1 min | ~ 100% | > 90% | [3] |
| Poly(methyl acrylate-co- methacrylic acid) (P(MA-MAA)) | None | Water environment | ~ 7 min | - | - | [4] |
| Vinyl-functionalized carbon dots (VCDs)/poly(acrylic acid-co-acrylamide) (P(AA-AAm))/3M VHB | None | Water environment | ~ 30 min | - | - | [5] |
| Poly([2-(methacryloyloxy)ethyl]trimethylammonium chloride-co-acrylic acid-co-2-hydroxyethyl methacrylate) (P(DMC-AA-HEMA))/*N*,*N*’-methylenebisacrylamide (MBAA) | None | HCl/NaCl solution environment | ~ 3 min | - | - | [6] |
| Poly(*N*-isopropylacrylamide) (PNIPAM)/*N*,*N*’-bis(acryloyl)-(l)-cystine salt (BISS)/Poly(vinyl alcohol) (PVA) | None | UV-assisted cold programming | < 7 min | ~ 80% | - | [7] |
| Poly(2-hydroxyethyl acrylate-co-isobornyl acrylate) (P(HEA-IBOA))/aliphatic urethane diacrylate (AUD) | None | Cold programming | ~ 1 min | > 95% | > 95% | [8] |
| Poly(acrylic acid-co-acrylamide) (P(AA-AAm))/poly(ethylene glycol) diacrylate (PEGDA)/sugar beet pulp (SBP)/glycerol | Sugar beet pulp (SBP) | Cold programming | < 1 min | > 90% | ~ 90% | Our work |

**Table S2.** Roughness of the SMPCs in the SMP-dominated and SBP-dominated regions.

| Roughness | SMP-dominated region | SBP-dominated region |
| --- | --- | --- |
| *Ra* (µm)  *Rq* (µm)  *Rz* (µm) | 0.0834  0.1101  0.4709 | 0.3995  0.5506  2.1831 |

**Table S3.** The compositions of the polymer resins with different contents of magnetised SBP.

| Content of SBP  (wt%) | AA  (g) | AAm  (g) | PEGDA  (g) | Glycerol  (g) | Water  (g) | SBP  (g) | Irgacure 819  (g) |
| --- | --- | --- | --- | --- | --- | --- | --- |
| 0  2.5  5.0 | 3.57  3.48  3.39 | 3.57  3.48  3.39 | 0.1  0.0975  0.095 | 1.09  1.063  1.035 | 1.76  1.716  1.672 | 0  0.25  0.5 | 0.145  0.141  0.138 |

**Reference**

[1] B. Zhang, H. Li, J. Cheng, H. Ye, A.H. Sakhaei, C. Yuan, P. Rao, Y.F. Zhang, Z. Chen, R. Wang, Mechanically robust and UV-curable shape-memory polymers for digital light processing based 4D printing. *Adv. Mater.* **2021**, *33*(27), 2101298.

[2] C.A. Spiegel, M. Hackner, V.P. Bothe, J.P. Spatz, E. Blasco, 4D printing of shape memory polymers: from macro to micro. *Adv. Funct. Mater.* **2022**, *32*(51), 2110580.

[3] C. Ni, D. Chen, Y. Yin, X. Wen, X. Chen, C. Yang, G. Chen, Z. Sun, J. Wen, Y. Jiao, Shape memory polymer with programmable recovery onset. *Nature* **2023**, *622*(7984), 748-753.

[4] W. Zhao, B. Wu, Z. Lei, P. Wu, Hydrogels with differentiated hydrogen-bonding networks for bioinspired stress response. *Angew. Chem. Int. Ed.* **2024**, *63*(21), e202400531.

[5] S. Wu, H. Shi, W. Lu, S. Wei, H. Shang, H. Liu, M. Si, X. Le, G. Yin, P. Theato, Aggregation-induced emissive carbon dots gels for octopus-inspired shape/color synergistically adjustable actuators. *Angew. Chem. Int. Ed.* **2021**, *60*(40), 21890-21898.

[6] G. Gao, K. Yin, J. Han, Y. Hu, J. Gu, J. Wei, T. Chen, Marine amoebae-inspired salting hydrogels to reconfigure anisotropy for reprogrammable shape morphing. *Angew. Chem. Int. Ed.* e202416672.

[7] C. Ni, D. Chen, X. Wen, B. Jin, Y. He, T. Xie, Q. Zhao, High speed underwater hydrogel robots with programmable motions powered by light. *Nat. Commun.* **2023**, *14*(1), 7672.

[8] L. Yue, X. Sun, L. Yu, M. Li, S.M. Montgomery, Y. Song, T. Nomura, M. Tanaka, H.J. Qi, Cold-programmed shape-morphing structures based on grayscale digital light processing 4D printing. *Nat. Commun.* **2023**, *14*(1), 5519.
